# Frank's sign with acute aortic dissection

**DOI:** 10.1002/ccr3.5625

**Published:** 2022-03-17

**Authors:** Hironobu Nishiori, Hiroyuki Watanabe, Yuichi Hirano, Masayoshi Otsu

**Affiliations:** ^1^ Division of Cardiovascular Surgery Narita Red Cross Hospital Chiba Japan

**Keywords:** aortic dissection, cardiovascular disease, coronary artery disease, Frank's sign

## Abstract

A 59‐year‐old man with a long smoking history presented with sudden back pain. Frank's sign was noticed in his bilateral ears, and computed tomography revealed Stanford type A acute aortic dissection. If young patients have Frank's sign, attention should be paid to aortic disease in addition to coronary artery disease.

## Graphical abstract

If young patients have Frank's sign, the possibility of aortic disease in addition to coronary artery disease needs to be recognized, and they should treat the risk factors to prevent future cardiovascular events.

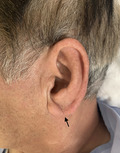

## 1 CASE

**FIGURE 1:**
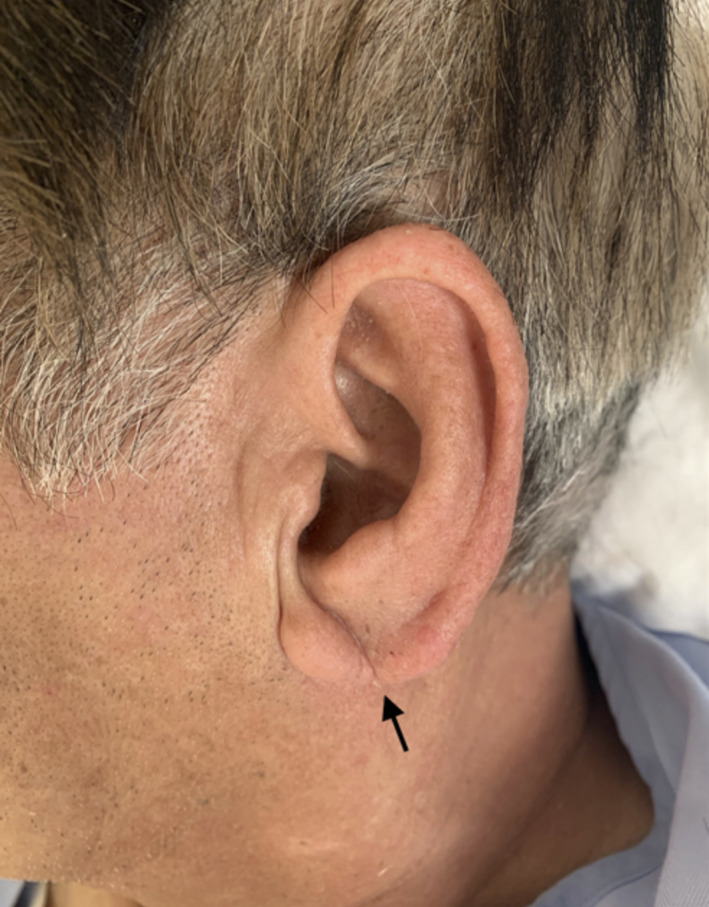
Frank's sign. A diagonal crease running across the earlobes at a 45° angle

**FIGURE 2:**
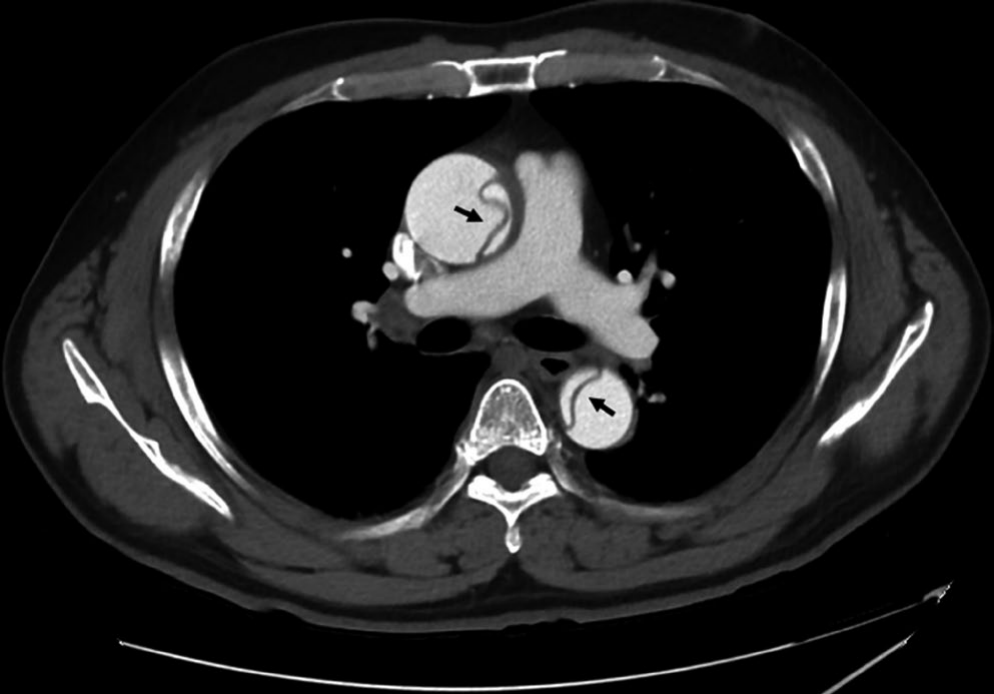
Computed tomography imaging showing the intimal flap in the aorta diagnosed as acute Stanford type A aortic dissection

**FIGURE 3:**
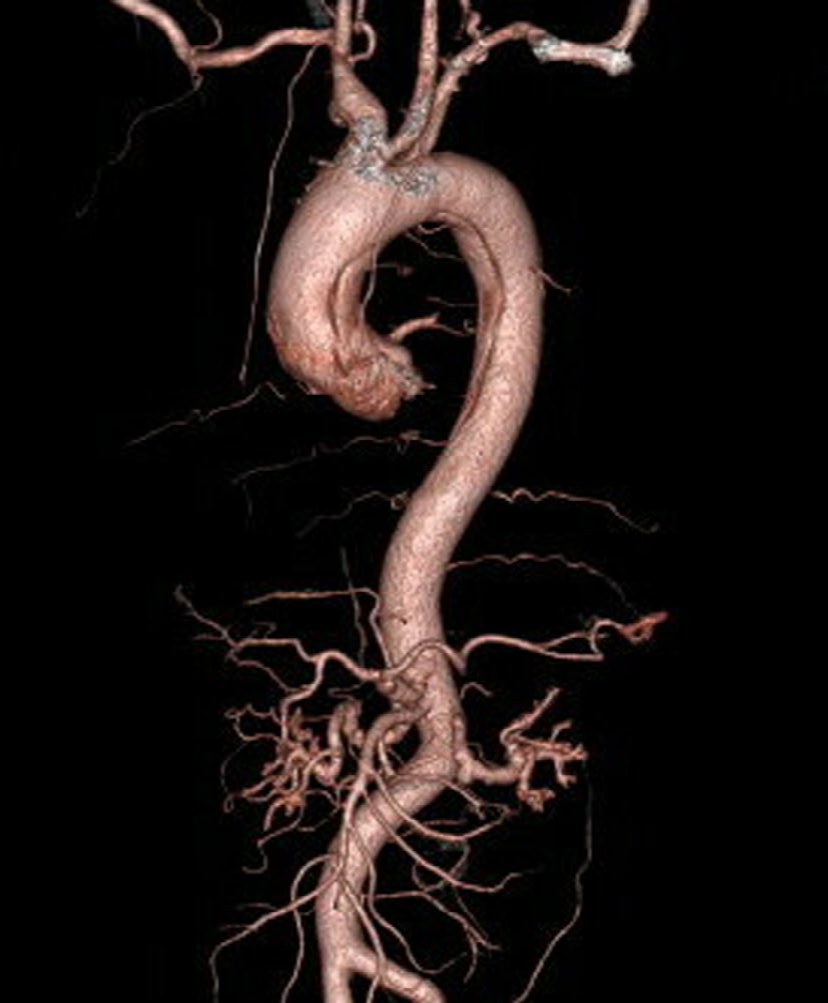
Preoperative 3D computed tomography imaging showed aortic dissection from ascending aorta to descending aorta

**FIGURE 4:**
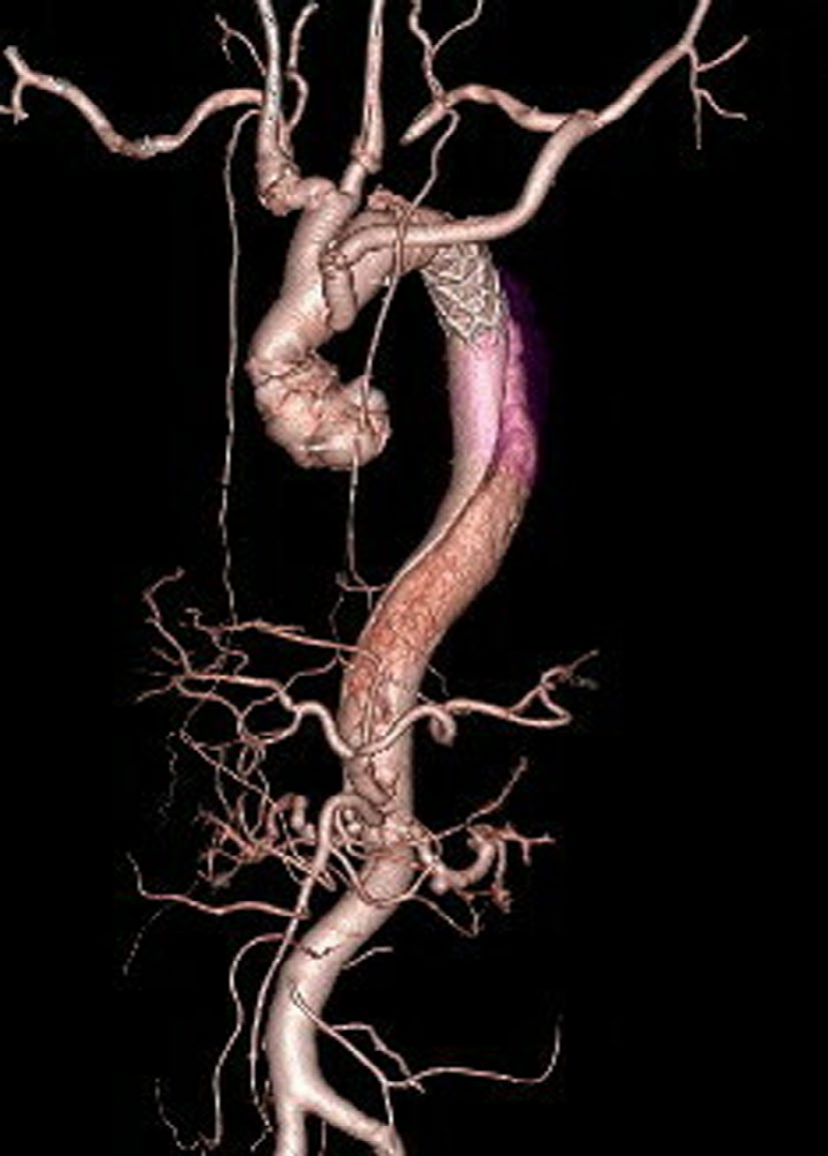
Post‐operative 3D computed tomography imaging showing ascending aorta and aortic arch replaced with an artificial graft

A 59‐year‐old man with a 40 pack‐year smoking history presented our hospital with sudden back pain. He had no remarkable past medical history. The blood pressure in the left arm was 30 mmHg higher than the right arm, and bilateral earlobes had a diagonal crease running across the earlobes at a 45° angle (Frank's sign; Figure 1). A contrasted computed tomography showed Stanford type A aortic dissection (Figure 2), and a 3D computed tomography also showed Stanford type A aortic dissection (Figure 3), whereas there was no significant calcification in the coronary arteries. The patient underwent total arch replacement for aortic dissection, and post‐operative 3D computed tomography showed the ascending aorta and aortic arch were replaced with artificial graft (Figure 4). Although he suffered from perioperative stroke with left arm paralysis, the symptom recovered, and he was transferred to another hospital for long‐term rehabilitation.

Frank's sign is caused by arteriosclerosis and is known to be associated with cardiovascular events, especially coronary artery disease, and should be paid more attention to when seen in people under the age of 60.^1^ Among the dead cases with Frank's sign, 12.3% were reported due to aortic disease.^2^ The factors contributing to atherosclerosis, such as hypertension and smoking, are also strong risk factors for aortic disease, including aortic dissection. For frank's sign in young people, in addition to coronary artery disease, the aortic disease should be noted.

## CONFLICT OF INTEREST

The authors have no pertinent conflict of interest to report for this manuscript.

## AUTHOR CONTRIBUTIONS

HN, YH, MO and HW cared for the patient and participated in the surgery. HN got the patient consent form and prepared the clinical picture and computed tomography imaging data, and wrote the report. HW read and approved the final version of the report.

## ETHICAL APPROVAL

None.

## CONSENT

Written informed consent was obtained from the patient to publish this report in accordance with the journal's patient consent policy.

## Data Availability

None.
